# Root Canal Treatment of Maxillary and Mandibular Three-Rooted Premolars: Case Reports

**Published:** 2012-08-01

**Authors:** Sousan Shalavi, Zahed Mohammadi, Maryam Abdolrazzaghi

**Affiliations:** 1. Hamedan University of Medical Sciences, Hamedan, Iran; 2. Department of Endodontics, Hamedan University of Medical Sciences, Hamedan, Iran; 3. Iranian Center for Endodontic Research, Shahid Beheshti University of Medical Sciences, Tehran, Iran; 4. Endodontist, Tehran, Iran

**Keywords:** Bicuspid, Dental Pulp Cavity, Mandible, Morphology, Root Canal, Tooth

## Abstract

Familiarity with the normal and abnormal anatomy of the root canal system is essential for successful root canal treatment. The possibility of concomitant three-rooted and three- canalled maxillary and mandibular premolars are extremely rare. The purpose of this paper was to report a case with a three-rooted maxillary first premolar and two three-rooted mandibular premolars.

## Introduction

A thorough knowledge of root canal morphology is essential to locate all the canals of a tooth root and to biomechanically treat and obturate in three dimensions [1]. Interestingly, premolar teeth show considerable variations in root canal morphology [2].

Maxillary premolars have highly variable root canal morphology. The maxillary first premolar typically has two well-formed roots (56%). These divide in the middle third of the root and lie buccal and lingual to one another. About 40% have only one root containing two canals (type IV) that then unite in a common foramen. Three- rooted maxillary first premolars are uncommon (0.5-6%) and frequently have one canal in each of three roots [1][2].

Lower incidence of three root canals (0.3%-2%) has been reported for second premolars [1].

The anatomy of maxillary premolars with three root canals, mesiobuccal, distobuccal and palatal, is similar to that of adjacent maxillary molars, and they are therefore sometimes called small molars or ridiculous [3].

Slowey has suggested that the mandibular premolars may present the greatest difficulty compared to other teeth when performing successful endodontic treatment [4].

Vertucci [5] described five different types of canal configuration for mandibular first premolar. Muller [6] reported that root canals in mandibular first premolars were usually quite round and conical, but inclined to be ribbon-like in the cervical third of the root and that wide buccolingual canals narrow into a bifurcation consisting of two very small canals [6]. Ingle et al. [7] described the shape of the canal as ovoid at the cervical level, round or ovoid at the mid root level and round at the apical third. The aim of this article was to report one case with a three-rooted maxillary first premolar and two cases with three-rooted mandibular premolars.

## Case Report

**Figure 1:**
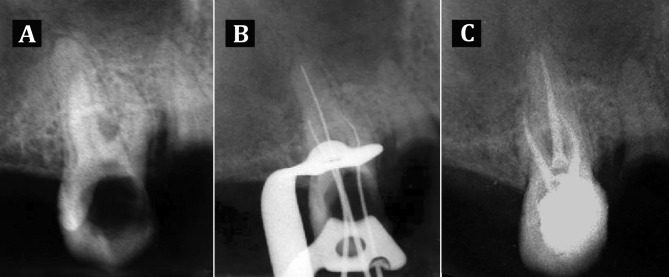
Periapical radiographs of upper right second premolar: A) Carious and radicular lesion; B) working length; C) post-operative radiograph

**Figure 2:**
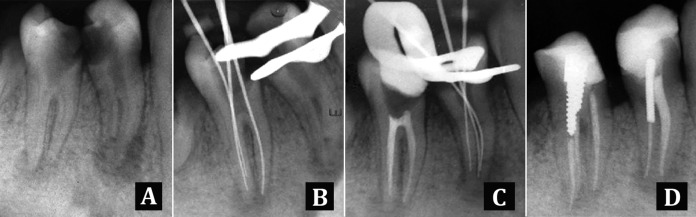
Periapical radiograph of lower premolars: A) Extensive caries and apical lesion; B) and C) working length; D) Final obturation

A 19 year old male was referred to the Department of Endodontics of Hamedan University of Medical Sciences for root canal treatment of tooth no. 5 (the upper right second premolar). His medical history was non-contributory. The patient’s chief complaint was pain in the right maxillary and left mandibular regions. Clinical examination showed large carious lesions in the upper right second and lower left first and second premolar teeth. Periapical radiographs confirmed the presence of large carious lesions (Figures 1A and 2A).

Due to the complexity of the root canal morphology, treatment was conducted in two separate sessions. The panoramic radiograph also showed that mandibular right premolar teeth were three-canalled.

First, the maxillary tooth was treated. The patient was given a local anesthetic infiltration using 2% lidocaine and 1:100,000 epinephrine (Darupakhsh, Tehran, Iran) and then tooth was isolated with rubber dam. After removing all caries, access cavity was prepared and working length of all three canals was determined (Figure 1B). All canals were prepared using Flex Master rotary files (VDW, Darmstadt, Germany) and 1.3% sodium hypochlorite irrigant (Golrang, Tehran, Iran). Thereafter, canals were dried with sterile paper points and were obturated with gutta-percha (AriaDent, Tehran, Iran) and AH-26 sealer (Dentsply, DeTrey, GmbH, Konstanz, Germany) using cold lateral compaction technique (Figure 1C). Finally the access cavity was filled with Coltosol (Aria Dent, Tehran, Iran).

The mandibular teeth were subsequently treated. After anesthetizing the teeth with inferior alveolar nerve block with the same local anesthetic, identical steps were taken (Figure 2B, 2C, 2D). Finally the access cavity was filled with Coltosol (AriaDent, Tehran, Iran).

**Figure 3:**
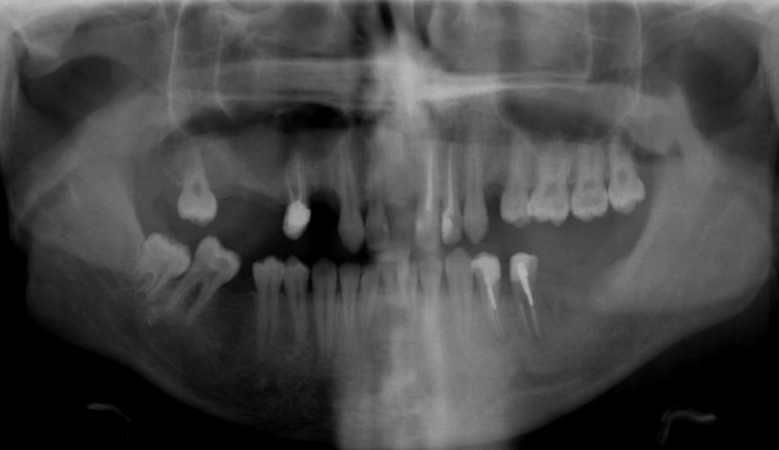
Dental Panoramic Tomograph of the patient after endodontic treatment

The panoramic radiograph (Figure 3) also illustrates coronal reconstruction of three endodontically treated premolars.

## Discussion

Clinically, precise three-dimensional determination of the internal structure of teeth, their form and number of root canals is a challenge. Each endodontic treatment is unique due to the high variability of the root canal system [3].

The possible anatomic configurations of maxillary premolars are well documented in literature. High quality preoperative radiographs and their careful examination are essential for the detection of additional root canals [4][8][9]. Maxillary first premolars usually have two canals. A third canal should be suspected clinically when the pulp chamber does not appear to be aligned in its expected bucco-palatal relationship. If the pulp chamber appears to deviate from normal configuration and seems to be either triangular in shape or too large in a mesiodistal plane, more than one root canal should be suspected [10]. In three rooted maxillary premolars, the buccal orifices are situated close to each other and therefore are hard to locate [3]. The outline of the access cavity was shaped with a bur at the bucco-proximal angle from the entrance of the buccal canals to the cavo-surface angle. In other words, outline of the access cavity is T-shaped [3].

Mandibular premolars usually have one root and one canal. However, 2-4 canals have also been reported [11][12]. Zillich and Dawson [13] showed that the incidence of three-rooted premolars was 0.04%. Rahimi et al. revealed that the incidence of three-canalled mandibular first premolars was 1.2% [14]. In a literature review, Cleghorn et al. (15) found that 0.2% of mandibular first premolars had three roots [11]. At least two radiographs, one right angle and the second at 15° to 20° angle mesial or distal from the horizontal long axis of the root, are required to reliably diagnose more than one root or root canal system [11]. Sudden narrowing of the main canal on a parallel radiograph was a good criterion to judge root canal multiplicity [15]. However, up to 40° mesial angulation from horizontal is required to reliably identifying the extra canals [16]. Deviation of the X-ray angle from the vertical axis of 15° to 30° was effective only in the mandibular first premolar in helping to visualize canal anatomy of premolar teeth. Dadresanfar et al. reported a two-rooted maxillary first premolar with two distinct canals in the apical third of buccal root (type IV) [17]. Furthermore, Asgary reported endodontic treatment of a mandibular second premolar with three separated canals in the apical third [18].

## Conclusion

Having thorough knowledge of root/root canal anatomy variations is essential for successful endodontic treatment.
